# Sialolipoma of the Floor of the Mouth with Immunohistological Analysis

**DOI:** 10.1155/2021/6623045

**Published:** 2021-04-05

**Authors:** Ahmed A. Zahrani, Ahmed Qannam, Ra'ed Al Sadhan, Ibrahim O. Bello

**Affiliations:** ^1^Department of Oral and Maxillofacial Surgery, King Saud University and University Dental Hospital, Riyadh, Saudi Arabia; ^2^Department of Oral Medicine and Diagnostic Sciences, King Saud University and University Dental Hospital, Riyadh, Saudi Arabia

## Abstract

Lipomas are relatively rare in the head and neck, and sialolipoma was described as an entity about 20 years ago as lipoma that entraps salivary gland tissue. Less than 10 cases have been described in the floor of the mouth not related to the major salivary glands. Here, we report a case of sialolipoma affecting the floor of the mouth in a 47-year-old patient and reviewed the clinical, histologic, and immunohistochemical characteristics of the lesion.

## 1. Introduction

Lipomas of the oral cavity are relatively very rare lesions, unlike their counterparts in the trunk and extremities where they constitute the most common mesenchymal tumor. Based on many reports, lipomas constitute between 1% and 5% of all benign oral lesions [1]. They are traditionally classified as fibrolipoma, spindle cell lipoma, angiolipoma, chondroid lipoma, intramuscular lipoma, and pleomorphic lipoma [2, 3]. A less frequently reported variant characterized by entrapment of salivary gland tissue containing acini and ducts by neoplastic mature adipocytes is called sialolipoma, a term that was first described by Nagao et al. [4] in 2001. Here, we report a case of sialolipoma of the floor of the mouth unrelated to the sublingual salivary gland.

## 2. Case Report

A 47-year-old Saudi male presented at the Oral and Maxillofacial Surgery Clinic of the University Dental Hospital at King Saud University Medical City (KSUMC) for assessment of swelling of the floor of the mouth reportedly of more than 3-year duration. The swelling was noted as a slow-growing, painless mass which caused the patient minimal discomfort and slight interference with speech, mastication, and movement of the tongue. The patient reported neither a history of trauma to the floor of the mouth nor that of intense pain while eating. The medical and family histories were noncontributory, and the patient was generally in good health.

**Figure 1:**
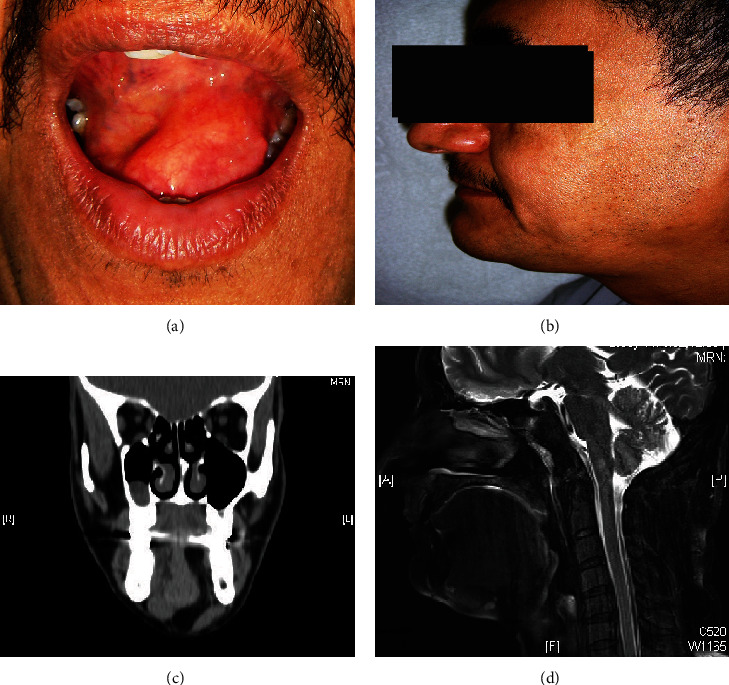
(a) Intraoral soft tissue mass in the floor of the mouth from the left ventral tongue surface extending beyond the midline to the right side. (b) Mild submental swelling as a result of an intraoral mass herniation beyond the mylohyoid muscle. (c) Coronal CT section at the level of the first molar in soft tissue window showing a markedly low density and clearly defined margin irregular mass within the left genioglossus muscle extending from ventral tongue to skin overlying the mylohyoid muscle. The density is homogenous and similar to adjacent subcutaneous fat. (d) Midsagittal MRI section in fat suppression showing the hypointense density mass.

Oral examination revealed the presence of a circumscribed, soft mass located on the left side of the oral cavity that was beside the tongue. The overlying mucosa was intact and appeared normal (Figures 1(a) and 1(b)). In its vertical dimension, when felt with bimanual palpation, the mass extended into the submental region. It caused a slight elevation of the tongue but no restriction of its movement. The salivary flow of the submandibular (Wharton) duct was normal, and there was no sensory deficit in the area. Although the patient reported occasional globus pharyngeus, there were no symptoms of nausea, dysphagia, snoring, or strident breathing. Lymph node examination was carried out for all neck nodes with no lymphadenopathy detected.

Imaging studies by CT scan and MRI showed an irregular mass in the area of the genioglossus muscle extending from the base of the tongue to the skin overlying the mylohyoid muscle with a density similar to the adjacent subcutaneous fat (Figure 1(c)). A hypointense density mass was observed in fat suppression MRI section at the level of the first molar suggesting an infiltrating intramuscular lipoma (Figure 1(d)). A tentative diagnosis of lipoma was made after clinical examination and radiographic examination, although the clinical differential diagnoses of the lesion included ranula, salivary gland neoplasm, and dermoid and epidermoid cyst.

**Figure 2:**
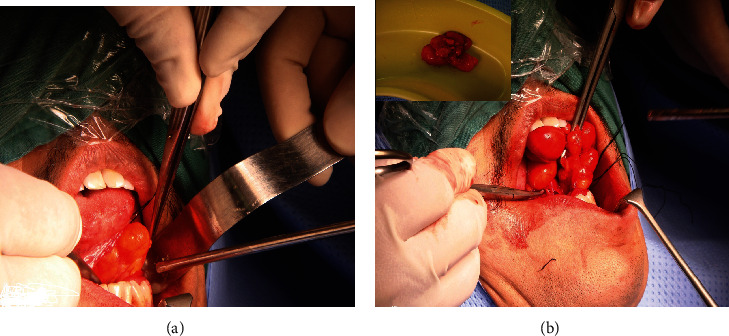
(a, b) Surgical excision of the intraoral mass. Mass was yellowish, soft, and irregular in shape. Inset in (b) is the whole mass after excision.

The patient underwent surgical treatment under general anesthesia, and the lesion was excised through an intraoral approach. The specimen was formed of lobulated spherical masses, yellowish to pale pinkish in color, and measured 3.5 × 4.7 × 1.5 cm in its greatest dimension (Figures 2(a) and 2(b)). The mass shelled out easily, with no adhesion to the sublingual gland or submandibular gland duct.

**Figure 3:**
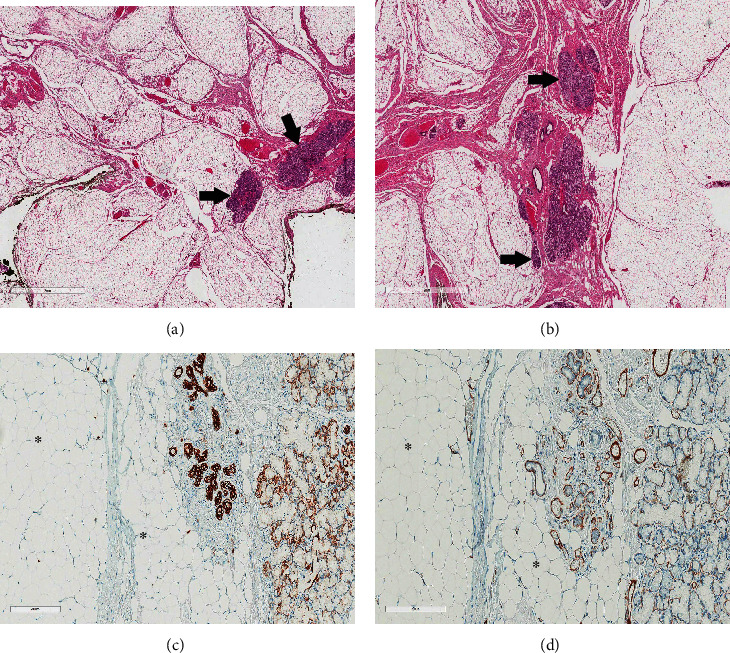
(a, b) Peripheral and inner areas of the tumor showing thin fibrous tissue capsule with numerous fat lobules and separated with fibrous tissue septae rich in vascular tissue with islands of salivary gland tissue (arrows) within the lipomatous proliferation. Scale bars: 2 mm. (c) Pancytokeratin (AE1/AE3) expression in the ductal and acinar cells in the entrapped salivary gland. The adipose tissue was negative (asterisk). Scale bar: 200 *μ*m. (d) Smooth muscle actin highlighting the myoepithelial cells around the acini and ducts, and the smooth muscle in adjacent vascular structures. Lipomatous elements were negative (asterisk). Scale bar: 200 *μ*m.

Histological examination showed an encapsulated lobulated tumor comprising mostly mature adipocytes. The lobules were separated by fibrovascular connective tissue septae in places. There were entrapped salivary gland acini and ducts scattered within the tumor mass (Figures 3(a) and 3(b)). The adipose tissue constituted about 90% of the tumor mass in comparison to the salivary gland tissue. A definitive diagnosis of sialolipoma was made. Immunohistochemical analysis of the tumor using pankeratin (AE1/AE3), epithelial membrane antigen (EMA), smooth muscle actin (SMA), S100, and Ki-67 immunostains was done. Acinar and ductal cells were strongly positive for AE1/AE3, luminal cells of ducts strongly expressed EMA alongside few acinar cells, and myoepithelial cells strongly stained by SMA and S100 with the latter also staining the fat cells. Proliferative activity as assessed by Ki-67 was less than 1% in both the adipose and salivary gland tissue (Table 1, Figures 3(c) and 3(d)).

The patient was followed up for more than 5 years, and there was no evidence of disease or recurrence.

## 3. Discussion

Sialolipoma was first described and reported as a series of 7 new cases in 2001 [4]. A subsequent review of 28 cases found in the literature as at 2009 showed that sialolipoma was seen in patients with a mean age of 52 years (range: 7 weeks to 84 years), occurred at relatively similar frequency in both males and females, and with most cases are seen in the parotid gland [5]. Sialolipoma of the minor salivary glands is very rare and is mostly found on the palate [4, 5]. Rare reports of cases occurring in the floor of the mouth have been reported mostly in females with all patients older than 60 years (Table 2) [6–9].

This report is that of a 47-year-old male patient with floor of the mouth sialolipoma, which coupled with its relatively large size is unusual for this specific entity. However, this is not a particularly rare finding in sialolipoma of other intraoral sites [9]. In general, this patient is younger than the mean age of presentation of sialolipomas [5]. Some reports have pointed to sialolipoma being more common in males [4, 10]. The lesion is generally characterized as being asymptomatic, slow-growing, soft, mobile, not associated with neurological deficits or mucosal or skin changes or other salivary gland lesions, or the presence of lipoma in other parts of the body [11]. All these features were seen in the present case. The occasional globus sensation reported by the patient could be attributed to the large size of the lesion and the elevation of the tongue as there was no pathology in the oropharynx, and this symptom was no longer present on follow-up after surgery.

Ultrasound examination would be the first choice among imaging investigations; however, lipomas may have a wide range of sonographic appearances [12]. The CT and MRI features are unique. On soft tissue window CT, it appears as a very low attenuation mass with minimal internal soft-tissue component [13]. MRI is the modality of choice for imaging lipomas, as it follows subcutaneous fat signal on all sequences appearing as high signal on T1 and saturates on fat-saturated sequences, with no or minimal contrast enhancement. On T2, it appears as a high signal on fast spin-echo sequences (FSE T2) and saturates on fat-saturated sequences [14].

Citing additional evidence from immunohistochemistry and ultrastructural studies, Nagao et al. [4] proposed that the salivary glandular elements seen in sialolipomas are non-neoplastic but rather became entrapped during the lipomatous proliferation. The glands are regularly organized and have normal phenotypes with little or no proliferative activity [4]. It is therefore assumed that the proliferative activity (which is relatively attenuated) resides in the adipocytes, although this is not demonstrable immunohistochemically. These features have been reaffirmed in other studies including a floor of mouth sialolipoma with some providing supportive immunohistochemical evidence for this hypothesis [6, 15], as in this present report. Microscopically, sialolipoma should be differentiated from salivary gland lipoadenoma (containing only dilated ducts along with fatty tissue but no salivary acini), lipomatous pleomorphic adenoma (containing chondromyxoid stroma), and salivary gland lipomatosis (unencapsulated proliferation of salivary acini, ducts, and mature adipocytes) [16, 17].

The treatment of choice is complete surgical removal as for all lipomas. In the present case in which the tumor arose in relation to minor salivary gland tissue in the floor of the mouth, there is no justification for removing the sublingual salivary gland as long as there is no proof that the tumor originates from the latter or found to be related to it during surgery. In the present case, the tumor shelled out without any adherence to the sublingual gland. Reported cases in the literature have shown no recurrence throughout the period of follow-up ranging from 7 to 91 months [5, 9, 10]. This patient was followed up for 62 months with no evidence of recurrence.

This report underlines that lipomas should be included in the differential diagnosis of asymptomatic, soft, mobile lesions of the floor of the mouth and that sialolipoma of the minor salivary glands could be the diagnosis found in microscopy after differentiation from relatively similar lesions such as lipoadenoma, lipomatosis, and pleomorphic adenoma with extensive lipomatosis. It may be more common than presently diagnosed as it is easy to overlook the presence of small scattered salivary gland elements. In addition, patients may be males younger than 60 years old.

## Figures and Tables

**Table 1:** Summary of immunohistochemical features of the floor of mouth sialolipoma.

Antibodies	Adipocytes	Acinar cells	Ductal cells	Antibody source
Epithelial membrane antigen (EMA)	—	+^∗^	+	Roche (Ventana)
Ki-67	—	—	—	Roche (Ventana)
Pankeratin (AE1/AE3)	—	+	+	Roche (Ventana)
S-100	+	+	—	Roche (Ventana)
Smooth muscle actin	—	+	+	Roche (Ventana)

^∗^Few acinar cell signals with bubbly positivity.

**Table 2:** Sialolipoma of the floor of the mouth.

Case	Author	Age	Sex	Duration	Size (cm)	Treatment	Follow-up
1	Lin et al. ^6^	67	F	1 year	3 × 2	Excision	NED 2 years
2	Leyva Huerta et al. ^7^	61	F	3 years	1.8 (diameter)	Excision	NA
3	Ponniah et al. ^8^	60	M	NS	2 (diameter)	Excision	NED 2 years
4	Nonaka et al. ^9^	73	F	NS	4 × 1	Excision	NA
5	Present case	47	M	3 years	4 × 5	Excision	NED 5 years

NA: not available; NED: no evidence of disease; NS: not specified.

## Data Availability

Data is restricted to King Saud University and not publicly available.
